# Antigen-Displaying Lipid-Enveloped PLGA Nanoparticles as Delivery Agents for a *Plasmodium vivax* Malaria Vaccine

**DOI:** 10.1371/journal.pone.0031472

**Published:** 2012-02-06

**Authors:** James J. Moon, Heikyung Suh, Mark E. Polhemus, Christian F. Ockenhouse, Anjali Yadava, Darrell J. Irvine

**Affiliations:** 1 Department of Materials Science and Engineering, Massachusetts Institute of Technology (MIT), Cambridge, Massachusetts, United States of America; 2 Department of Biological Engineering, Massachusetts Institute of Technology (MIT), Cambridge, Massachusetts, United States of America; 3 Division of Malaria Vaccine Development, Walter Reed Army Institute of Research, Silver Spring, Maryland, United States of America; 4 Koch Institute for Integrative Cancer Research, Massachusetts Institute of Technology (MIT), Cambridge, Massachusetts, United States of America; 5 Ragon Institute of MGH, MIT, and Harvard, Boston, Massachusetts, United States of America; 6 Howard Hughes Medical Institute, Chevy Chase, Maryland, United States of America; Universidade Federal de Minas Gerais, Brazil

## Abstract

The parasite *Plasmodium vivax* is the most frequent cause of malaria outside of sub-Saharan Africa, but efforts to develop viable vaccines against *P. vivax* so far have been inadequate. We recently developed pathogen-mimicking polymeric vaccine nanoparticles composed of the FDA-approved biodegradable polymer poly(lactide-*co*-glycolide) acid (PLGA) “enveloped” by a lipid membrane. In this study, we sought to determine whether this vaccine delivery platform could be applied to enhance the immune response against *P. vivax* sporozoites. A candidate malaria antigen, VMP001, was conjugated to the lipid membrane of the particles, and an immunostimulatory molecule, monophosphoryl lipid A (MPLA), was incorporated into the lipid membranes, creating pathogen-mimicking nanoparticle vaccines (VMP001-NPs). Vaccination with VMP001-NPs promoted germinal center formation and elicited durable antigen-specific antibodies with significantly higher titers and more balanced Th1/Th2 responses *in vivo*, compared with vaccines composed of soluble protein mixed with MPLA. Antibodies raised by NP vaccinations also exhibited enhanced avidity and affinity toward the domains within the circumsporozoite protein implicated in protection and were able to agglutinate live *P. vivax* sporozoites. These results demonstrate that these VMP001-NPs are promising vaccines candidates that may elicit protective immunity against *P. vivax* sporozoites.

## Introduction

*Plasmodium vivax* is the most frequent cause of malaria outside of sub-Saharan Africa and infects up to 390 million people each year [1]. Despite its heavy burden on global health and potential for spread outside of its endemic regions, *P. vivax* has not received as much attention from the vaccinology community as *P. falciparum*, a more virulent malaria strain [2]. However, recent studies have suggested that the impact of *P. vivax* has been underestimated and its public health burden is on the rise [3]. To address the lack of potential vaccine candidates for *P. vivax*, we recently developed *vivax* malaria protein (VMP001), a recombinant antigen derived from the circumsporozoite protein (CSP), the most prevalent membrane protein on sporozoites [4], [5]. Our prior studies have shown that VMP001 mixed with conventional adjuvants (e.g., Montanide) can elicit VMP001-specific antibody responses [4], [5]. However, as shown in previous clinical trials, elicitation of protective immunity against malaria sporozoites may require more potent adjuvants that generate durable humoral immune responses with increased avidity and affinity toward the CSP, especially against potentially protective epitopes [6].

Robust antibody responses characterized by longevity and high avidity require activation of B cells, followed by their affinity maturation and differentiation into memory B cells and long-lived plasma cells. To activate B cells, cell surface B-cell receptors (BCRs) need to be crosslinked by binding to cognate epitopes in antigen in a multivalent manner, as presented on the surfaces of foreign pathogens [7], [8]. Taking design cues from viral/bacterial pathogens, many research groups have devised particulate vaccines that can display repeat copies of antigens on the surfaces of particles, thus enhancing activation of B cells and humoral immune responses [9], [10], [11], [12], [13], [14]. In addition, particle vaccines can be loaded with “danger” signals that trigger Toll-like receptors (TLRs) or NOD-like receptors (NLRs) in B cells and dendritic cells, thereby eliciting robust humoral immune responses [9], [15], [16], [17].

We recently reported the development of pathogen-mimicking polymeric vaccine nanoparticles and microparticles, based on a core of the FDA-approved biodegradable polymer poly(lactide-*co*-glycolide) acid (PLGA) “enveloped” by a lipid membrane [18], [19]. These particles were prepared by emulsifying an organic phase of polymer and co-dissolved lipids in water, leading to self-assembled lipid coatings surrounding each particle [18]. PLGA particles can activate the inflammasome in antigen-presenting cells via NLRP3 and enhance innate and adaptive immune responses [20], [21]. At the particle surface, the lipid bilayers surrounding this PLGA core can be incorporated with lipophilic pathogen-associated molecular patterns (PAMPs) and protein antigens, creating particles with a pathogen-mimetic composition of repeated antigen and PAMP motifs [19]. Using this strategy, we co-delivered the Toll-like receptor agonist monophosphoryl lipid A (MPLA) and/or the natural killer T-cell ligand α-galactosyl ceramide together with the model protein ovalbumin (OVA) bound to lipid-enveloped particles with bacteria- or virus-like sizes (∼2.5 µm or ∼250 nm diam., respectively), and showed that these pathogen-mimicking particles can generate strong antigen-specific humoral and cellular immune responses *in vivo* [19].

To determine whether this novel vaccine delivery platform using FDA-approved materials could be used to enhance the immune response against our candidate vivax antigen, here we synthesized lipid-enveloped PLGA NPs displaying the VMP001 malaria antigen (VMP001-NPs), and tested their efficacy in generation of antigen-specific humoral immune responses *in vivo*. Vaccination with VMP001-NPs led to germinal center (GC) formation and elicited durable antigen-specific antibodies with significantly higher titers and more balanced Th1/Th2 responses, compared with soluble protein vaccines. Antibodies elicited with NP vaccines also exhibited enhanced avidity and affinity toward the domains within CSP implicated in protection and, ultimately, had the capacity to agglutinate live *P. vivax* sporozoites, suggesting that these NP vaccines may elicit protective immunity in field clinical trials.

## Materials and Methods

### Materials

PLGA with a 50∶50 lactide∶glycolide ratio was purchased from Lakeshore Biomaterials (Birmingham, AL). The lipids 1,2-dioleoyl-sn-glycero-3-phosphocholine (DOPC), 1,2-dioleoyl-sn-glycero-3-phospho-(1′-rac-glycerol) (DOPG), and 1,2-distearoyl-sn-glycero-3-phosphoethanolamine-N-[maleimide] (mal-PE) were purchased from Avanti Polar Lipids (Alabaster, AL). MPLA was purchased from Sigma Aldrich (St. Louis, MO). *n*-succinimidyl s-acetyl(thiotetraethylene glycol) (SAT(EG)_4_), 3,3′,5,5′ tetramethylbenzidine (TMB), strepavidin-coated ELISA plates, and 7000 MWCO desalting spin columns were from Pierce Biotechnology (Rockford, IL). PEG-thiol (2 kMW) was purchased from Laysan Bio (Arab, AL). The Lavapep Protein Quantification Assay was obtained from Gel Company (San Francisco, CA). Horseradish peroxidase (HRP)-labeled anti-mouse IgG, IgG1, IgG2b, IgG2c, and IgG3 were purchased from Invitrogen (Carlsbad, CA). Anti-B220, anti-IgD, anti-GL-7, and anti-goat IgG conjugated with Alexa fluor 488 were obtained from BD Bioscience (Franklin Lakes, NJ). Goat anti-his-tag IgG was obtained from Abcam (Cambridge, UK) and peanut agglutinin was from Vectorlabs (Burlingame, CA). Peptide epitope sequences were synthesized by the Koch Institute Biopolymers Facility at MIT (Cambridge, MA). All other reagents and solvents were obtained from Sigma Aldrich and used as received unless noted otherwise.

### Lipid-enveloped nanoparticle synthesis and characterization

Lipid bilayer-enveloped nanoparticles were synthesized as previously reported [18], [19]. Briefly, a lipid solution containing DOPC, DOPG, and mal-PE in a 4∶1∶5 molar ratio were do-dissolved with 30 mg PLGA polymer (50∶50 lactide∶glycolide ratio) in dichloromethane (DCM). In this organic phase, 200 µl PBS was dispersed by sonication for 1 minute on ice using a Misonix XL2000 Probe Tip Sonicator (Farmingdale, NY) at 7 W output power. The resulting solution was immediately dispersed in 6 mL distilled deionized (DDI) water by sonication for 3.5 min on ice using the Misonix XL2000 at 12 W output power. DCM was evaporated overnight at 25°C while agitating the solution on an orbital shaker. To purify polymer-core nanoparticles from free liposomes, particles were layered over a cushion of 30% sucrose in ultrapure water and centrifuged at 13,000×*g* for 5 minutes. The liposome-containing solution retained above the sucrose gradient was discarded, and the particles pelleted below the sucrose gradient were washed twice in PBS with centrifugation at 6000×*g* for 5 minutes. The PLGA particles were subsequently centrifuged at 50×*g* for 1 minute to remove large aggregates, and the supernatant containing nanoparticles were used for conjugation with VMP001 antigen. Particle sizes were determined by dynamic light scattering (DLS) using a 90Plus/ZetaPals particle size (Brookhaven Instruments) and confirmed with the Horiba Partica LA-950V2 Laser Diffraction Particle Size Analysis System. SEM images of particles were obtained by drying particles onto a substrate, coating the dried particles with 15 nm of Au to using an ion beam sputter coater (Gatan, Pleasanton, CA), and imaging the samples using an FEI/Philips XL30 FEG ESEM with 15 kV accelerating voltage.

### VMP001 antigen

The design and production of VMP001 has been reported previously [4], [5]. Briefly, the Vivax Malaria Protein 001 represents the circumsporozoite protein of *P. vivax.* It is comprised of a central repeat region, encoding the repeat motifs of two major subtypes of *P. vivax*, flanking the N- and C-terminal regions of the protein. The antigen was cloned and expressed in *E. coli* and purified using affinity and ion-exchange chromatography. It was tested to be free of host contaminants, including endotoxin.

### Conjugation of VMP001 to lipid-enveloped particles

To surface-display VMP001 on lipid-enveloped particles, thiolated VMP001 was linked via maleimide-functionalized-PE in the particle lipid coatings. First, VMP001 was modified with the heterobifunctional cross-linker *n*-succinimidyl s-acetyl(thiotetraethylene glycol) (SAT(EG)_4_) by adding a 10-fold molar excess of the crosslinker (2.2 mM) to VMP001 solution (0.22 mM or 10 mg/mL) and incubating on a revolving rotator for 30 min at 25°C. To quench NHS groups on unreacted SAT(EG)_4_ molecules, 25 mM glycine was added, and the protein was incubated for an additional 15 min rotating at 25°C. Quenched SAT(EG)_4_ was removed by buffer exchange with a 7000 MWCO desalting spin column. Sulfhydryl groups on SAT(EG)_4_-modified VMP001 were deprotected by adding 50 mM hydroxylamine and 2.5 mM EDTA (pH = 7.4) and rotating for 2 hr at 25°C followed by a second buffer exchange into 10 mM EDTA (pH = 7.4). PLGA particles were incubated with thiolated VMP001 (260 µg protein per mg of PLGA NPs) for 2 hrs at 25°C before washing with sterile saline to remove unbound antigen. PLGA particles conjugated with VMP001 were then incubated with 2 kMW PEG-thiol at 10 mg/ml for 1 hr at 37°C before washing with sterile saline to remove unbound PEG-thiol. The dose of protein on PEGylated VMP001-NPs was determined by two methods. VMP001 was stripped from particles using 0.2% Triton X-100 and the released protein was quantified by direct fluorescence measurement (using VMP001 labeled with Alexa Fluor 555), and also using the Lavapep Protein Quantification Assay. Surface-exposed VMP001 on NPs was visualized with confocal microscopy (LSM510, Carl Zeiss, Germany) after incubating VMP001-NPs with polyclonal goat anti-his-tag antibody and Alexa-fluor 488 labeled anti-goat IgG.

### Immunizations

Mice were cared for in the USDA-inspected MIT Animal Facility under federal, state, local and NIH guidelines for animal care. All protocols for in vivo experiments were approved by the Institutional Animal Care and Use Committee at the Massachusetts Institute of Technology (Approval number: 0708-074-11). Groups of six- to ten-week old female C57Bl/6 mice (Jackson Laboratories, Bar Harbor, ME) were immunized *s.c.* at the tail base with 100 µL of VMP001 either in soluble or PLGA NP formulations in PBS on days 0 and 21. The tail base site was chosen based on pilot experiments comparing *s.c.* flank and tail base immunizations, where tail base *s.c.* sites elicited ∼10-fold higher antibody responses in all groups (data not shown). Doses chosen for these studies were motivated by our initial studies using Freund's adjuvant or Montanide [4], [5], where we found 1–10 µg of VMP001 elicited substantial IgG titers in mice. Indicated doses of TLR-4 agonist, MPLA, were admixed either to soluble VMP001 or VMP001-NP prior to both prime and boost immunizations.

### Characterization of anti-VMP001 sera

At various time points, sera were collected and tested for anti-VMP001 antibodies by ELISA [5]. Briefly, 96-well plates coated with 0.04 µg of VMP001 in PBS at 25°C overnight were washed with PBS-Tween20, and blocked with 0.5% casein in PBS for an hour. The plates were incubated with sera diluted in 0.5% casein buffer for 2 hr at 25°C, developed using horseradish peroxidase (HRP)-labeled anti-mouse IgG, IgG1, IgG2b, IgG2c, or IgG3 and 3,3′,5,5′ tetramethylbenzidine (TMB), and read at A_450_. Anti-VMP001 serum titers were defined as the lowest sera dilution at which optical density (OD) reading was ≥0.5. Avidity measurements were performed by adding an extra step of incubation with 6 M urea for 10 min at 25°C and washing prior to incubation with anti-mouse IgG antibody to remove weakly bound IgG from plates [22]. The avidity index was defined as:
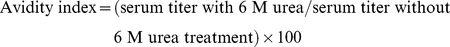
For epitope analysis of sera, streptavidin-coated ELISA plates were incubated with 50 µg/ml of the following epitopes from VMP001: Type-1 repeat (GDRAAGQPAGDRADGQPA), AGDRx5 (AGDRAGDRAGDRAGDRAGDR), Region I (NPRENKLKQP), Region II (EWTPCSVTCGVGVRVRRR), C-terminus (PNEKSVKEYLDK), and a scrambled peptide control (KPLDVEKNSEY), each synthesized with biotin-GSSSG as spacer at the N-terminus. After 12 hr incubation at 4°C, anti-VMP001 IgG titer measurements were performed as described above for VMP001-coated plates.

### Cytokine analysis

C57Bl/6 mice were vaccinated on days 0 and 21 with 1 **µ**g VMP001 and alum or with 5 **µ**g MPLA in either soluble or PLGA formulations, and splenocytes isolated on day 28 were stimulated with 25 **µ**g/ml VMP001 *ex vivo*. After 2 days, cytokine levels in the culture supernatants were measured by flow cytometry-based multiplex cytokine analysis kit (Cytometric Bead Array, BD).

### Germinal center analysis

On day 21 after immunization with 0.1 **µ**g VMP001 either in soluble or PLGA NP formulations and 5 **µ**g MPLA *s.c.* at tail base, draining inguinal lymph nodes were isolated, stained with markers for GCs, anti-B220, anti-IgD, anti-GL-7, peanut agglutinin, and analyzed with flow cytometry. To visualize GCs in draining inguinal lymph nodes, cryosectioned lymph nodes were fixed in 4% paraformaldehyde, blocked with 1% BSA, stained with anti-B220, anti-IgD, and anti-GL-7, and visualized with confocal microscopy.

### Live *P. vivax* sporozoite immunofluorescence assay

*Anopheles dirus* mosquitoes were fed with blood collected from *P. vivax*-infected patients. Sporozoites were obtained from the salivary glands of infected mosquitoes approximately 17 to 21 days after the blood meal and typed for the VK210 subtype of *P. vivax*. Sporozoites were washed with PBS and then incubated with equal volumes of mouse anti-VMP001 sera for 30 min. Anti-mouse IgG-fluorescein isothiocyanate diluted 1∶40 in PBS–0.1% BSA was added to the slide, and after 30 min the slides were observed by fluorescence microscopy at 40× magnification.

### Statistical analysis

Statistical analyses were carried out using GraphPad Prism 5.0c software. Student's *t* test was used to compare two groups. Two-way analysis of variance (ANOVA), followed by a Bonferroni post-test was used to compare >2 groups. *p*-values less than 0.05 were considered statistically significant. All values are reported as mean ± s.e.m.

## Results

### Synthesis of lipid-enveloped PLGA NPs displaying malaria antigen and adjuvant

**Figure 1:**
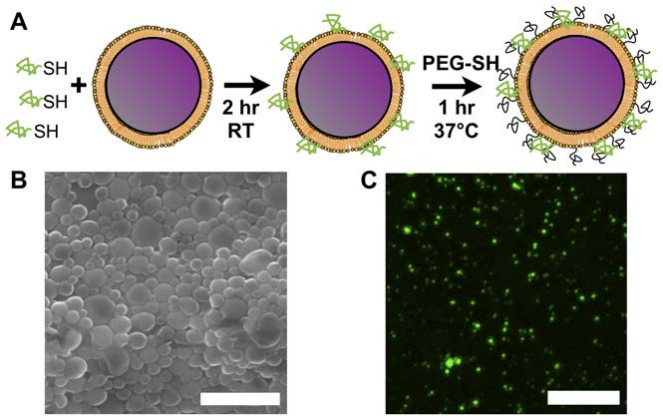
Synthesis of PLGA NPs with surface-conjugated VMP001 (VMP001-NPs). (A) Schematic illustration of synthesis of lipid-enveloped PLGA NPs with surface-conjugated VMP001. PLGA NPs were incubated with thiolated VMP001, conjugating the antigen to maleimide-functionalized lipids displayed on the particle membranes. Particles were then PEGylated in a reaction with PEG-thiol. (B) A scanning electron microcopy image of VMP001-NPs. Scale bar, 2 µm. (C) Confocal microscopy image of VMP001-NPs incubated with anti-his-tag and fluorescent secondary antibodies to detect particle surface-conjugated VMP001. Scale bar, 10 µm.

Lipid-enveloped PLGA NPs were synthesized by a W∶O∶W double-emulsion/solvent evaporation method, with PLGA and lipids (DOPC:DOPG:maleimide-headgroup phosphoethanolamine in a 4∶1∶5 mol ratio) co-dissolved in the organic phase. Self-assembly of lipid at the surface of each NP allowed subsequent conjugation of thiolated VMP001 to maleimide groups displayed in the lipid coating of the particle surfaces (**Fig. 1A**). In a typical VMP001-NP synthesis, 26±3.2 µg of VMP001 was conjugated per mg of lipid-coated PLGA with 10±2% protein conjugation efficiency, as measured with fluorophore-tagged VMP001 and independently by a commercial fluorescence-based protein quantification assay. After antigen conjugation, the percentage of remaining reactive maleimide groups on the particles decreased to 26±7.6%; the remaining reactive groups were quenched by coupling with 2 KDa thiol-terminated PEG. VMP001-NPs examined under a scanning electron microscope showed a relatively homogenous particle size distribution (**Fig. 1B**). Dynamic light scattering measurements indicated that PEGylated, VMP001-displaying PLGA NPs (VMP001-NPs) had a mean hydrodynamic diameter of 290 nm. To confirm that VMP001 was conjugated on the membranes of particles, we probed for surface-exposed VMP001 bound to the NPs using antibodies against the C-terminal his-tag of the VMP001 protein. VMP001-NPs incubated with anti-his-tag antibodies and fluorophore-conjugated secondary antibodies showed antibody binding to the particles (**Fig. 1C**) while control particles without VMP001 showed no staining (not shown), indicating that the antigen was linked to the membranes of VMP001-NPs.

### Vaccination with VMP001-NP induces durable antibody responses at 10-fold lower doses of antigen than soluble protein

**Figure 2:**
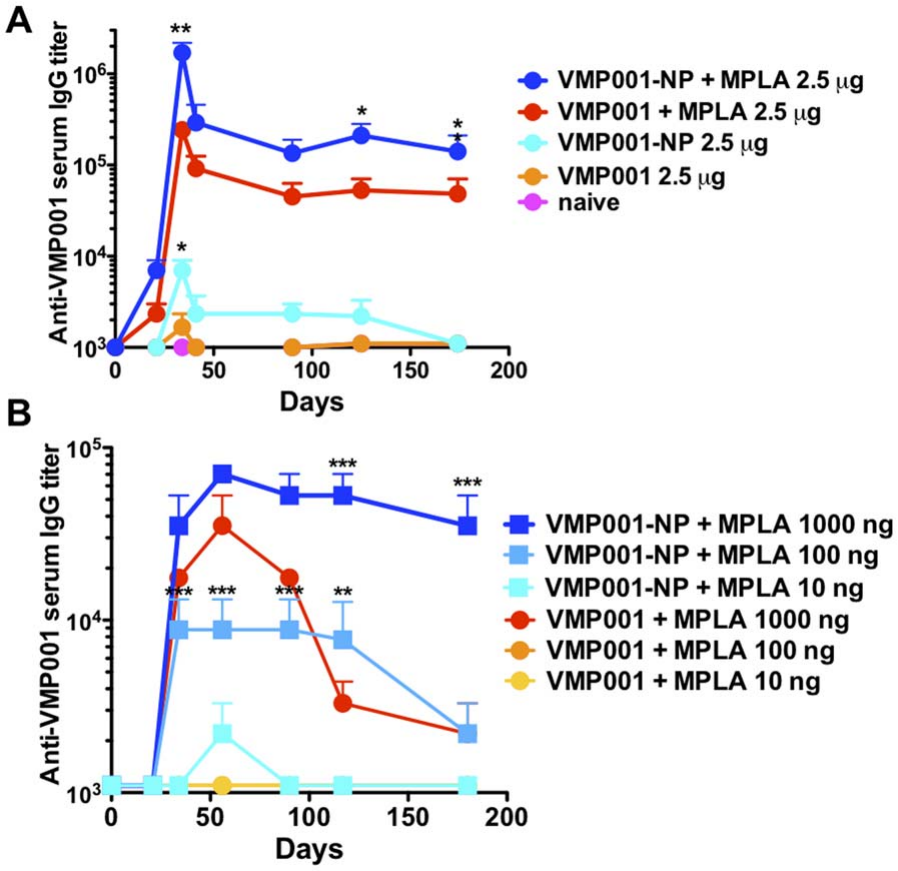
VMP001-NP vaccination mediates potent humoral immune responses. (A) C57Bl/6 mice were immunized *s.c.* on days 0 and 21 with 2.5 µg of VMP001 in either soluble or VMP001-NP formulations, with or without the addition of 25 µg MPLA. Anti-VMP001 IgG serum titer were monitored over time by ELISA. (B) C57Bl/6 mice were immunized *s.c.* on days 0 and 21 with the indicated doses of VMP001 in either soluble or VMP001-NP formulations with the fixed dose of 25 µg MPLA admixed in all groups. *, *p*<0.05, **, *p*<0.01, and ***, *p*<0.001, for VMP001-NP formulations compared to soluble VMP001 vaccines at the same dose of antigen, analyzed by two-way ANOVA followed by Bonferroni post-tests.

We first investigated the effect of conjugating VMP001 to particles by evaluating the humoral immune responses against the antigen in the presence or absence of MPLA, a TLR-4 agonist. C57Bl/6 mice were immunized on days 0 and 21 with 2.5 µg of VMP001-NP or soluble VMP001, with or without the addition of 25 µg MPLA. Serum VMP001-specific IgG titers were measured over time by ELISA. Vaccination with VMP001-NP generated higher titers than soluble VMP001. However, addition of MPLA to both vaccine formulations resulted in significantly elevated VMP001-specific IgG titers that were maintained for over 6 months (**Fig. 2A**). In particular, vaccinations with VMP001-NP+MPLA elicited significantly higher antibody titers compared with soluble VMP001+MPLA vaccines (days 35, 125 and 174 with *p*<0.001, *p*<0.05, and *p*<0.05, **Fig. 2A**). Based on these results, our subsequent immunization studies were all performed with vaccines mixed with MPLA.

To further delineate the effect of delivering VMP001 on lipid-shell PLGA NPs, mice were immunized with titrated doses of VMP001 in NP or soluble formulations. Immunizations with VMP001-NP mixed with 25 µg MPLA elicited high titers of serum IgG against the antigen with as little as 100 ng of antigen, which were maintained for more than 6 months (**Fig. 2B**). In contrast, vaccines composed of soluble protein mixed with 25 µg MPLA required at least 10-fold more antigen to elicit a response, and the titers achieved were maintained only transiently, with titers waning rapidly after reaching a peak by day 56.

**Figure 3:**
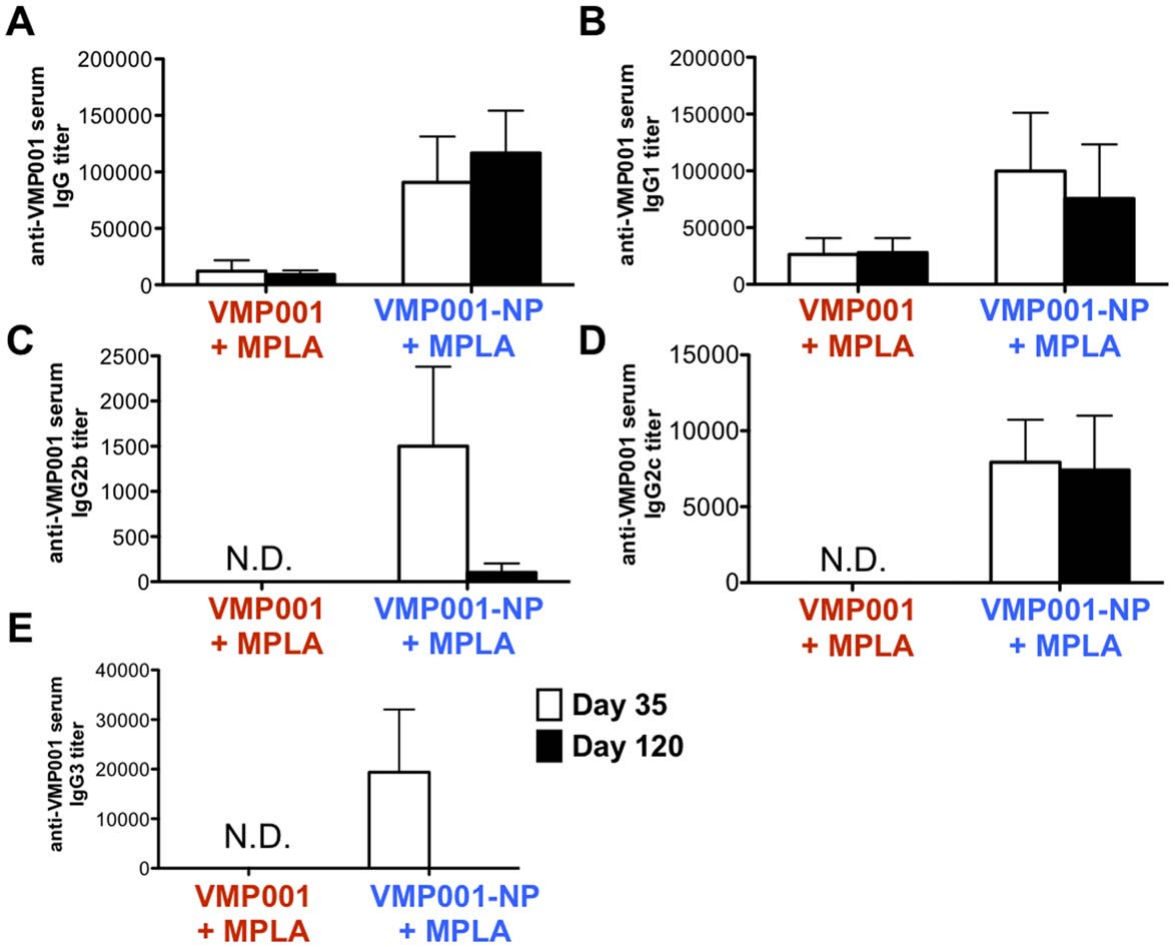
Immunization with VMP001-NPs elicits Th1/Th2 balanced antibody responses. (A) C57Bl/6 mice were immunized *s.c.* on days 0 and 21 with 25 µg of MPLA and 1 µg of VMP001 in either soluble or VMP001-NP formulations, and anti-VMP001 IgG sera were characterized on days 35 and 120 for (A) IgG, (B) IgG_1_, (C) IgG_2b_, (D) IgG_2c_, and (E) IgG_3_ titers.

**Figure 4:**
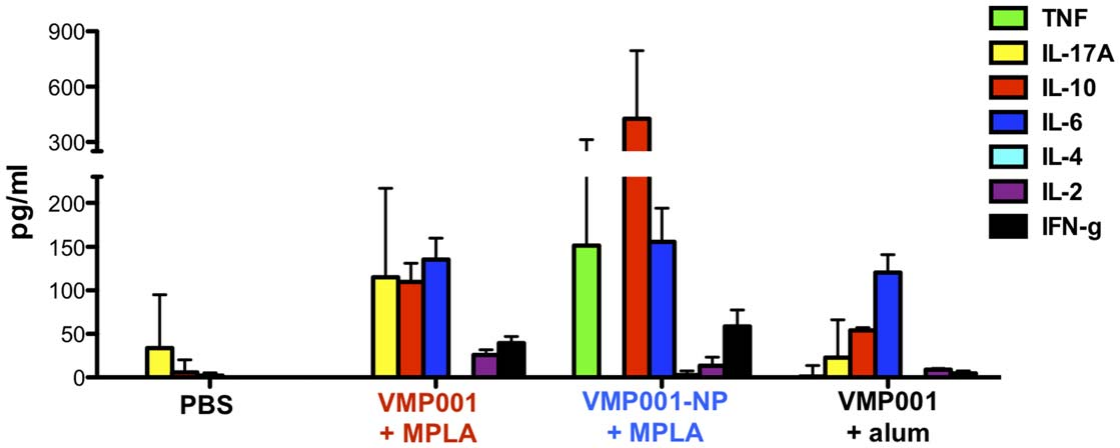
Vaccination with VMP001-NPs enhances cellular immune responses. C57Bl/6 mice were vaccinated on days 0 and 21 with 1 µg VMP001 and alum or with 5 µg MPLA in either soluble or VMP001-NP formulations, and splenocytes isolated on day 28 were stimulated with 25 µg/ml VMP001 *ex vivo*. After 2 days, the supernatants were assayed for the presence of cytokines by ELISA.

Sera from mice immunized and boosted with 1 µg of antigen were obtained on days 35 and 120 post immunization and further analyzed to determine the isotypes of antibodies generated by soluble VMP001 vs. VMP001-NP immunization. At the specified antigen dose, the nanoparticle vaccines promoted a more balanced Th1/Th2 antibody response, with sustained IgG_1_ and IgG_2c_ responses and transient elicitation of IgG_2b_ and IgG_3_ VMP001-specific antibodies whereas soluble protein+MPLA generated Th2-skewed, weak IgG_1_ antibody responses (**Fig. 3A–E**). Although clear difference in the Th1/Th2 balance of the immune response were detected in the antibody isotype analysis, measurement of cytokine production by restimulated splenocytes *ex vivo* did not reveal statistically significant differences in VMP001-NP, soluble VMP001, or alum-immunized groups (**Fig. 4**).

### Enhanced germinal center formation promoted by NP vaccination

**Figure 5:**
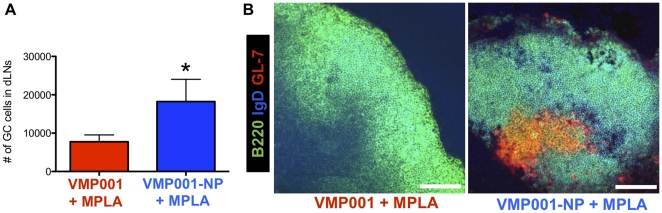
VMP001-NP immunization triggers germinal center formation. (A) C57Bl/6 mice were vaccinated with 0.1 µg VMP001 and 5 µg MPLA in either soluble or VMP001-NP formulations, and on day 21, inguinal dLNs were isolated and analyzed for germinal center formation. The number of isotype-switched germinal center B cells (GL-7^+^PNA^+^), gated on B220^+^IgD^low^ populations in dLNs was measured with flow cytometric analysis. (B) Inguinal dLNs were cryo-sectioned on day 21 post-immunization and stained with anti-B220, anti-IgD, and anti-GL-7 (markers for B cells, immature B cells, and germinal center, respectively) and examined by confocal microscopy. Scale bars, 50 µm. *, *p*<0.05, analyzed by Student's *t* test.

Long-lived, high titer humoral responses elicited by NP immunizations suggest that the particle vaccine may promote formation of germinal centers (GCs), which are specialized regions in lymphoid organs where activated B cells proliferate and mature to form long-lived memory B cells [23], [24], [25]. Prior studies of NP vaccines have shown that the vast majority of particles accumulate in draining lymph nodes and this is the site where GC reactions occur [17]. Thus, to test the relative potency of soluble vs. VMP001-NP vaccines to promote GC formation, we immunized mice with a limiting dose of 5 µg MPLA and 0.1 µg soluble or VMP001-NP and analyzed the response in draining lymph nodes (dLNs). Flow cytometry analysis of dLNs on day 21 revealed that VMP001-NP vaccines induced ∼2.5-fold greater numbers of isotype-switched GC B-cells (B220^+^IgD^low^GL-7^+^PNA^+^), compared to soluble VMP001+MPLA vaccines (p<0.05, **Fig. 5A**). In addition, immunohistochemical analysis of dLNs on day 21 showed that even this very low dose of antigen when delivered by NPs promoted recognizable GL-7^+^ germinal center formation, whereas organized GCs were absent in mice immunized with the same doses of soluble protein mixed with MPLA (**Fig. 5B**).

### VMP001-NP generates high affinity antibodies against the repeat domains of the CS antigen

**Figure 6:**
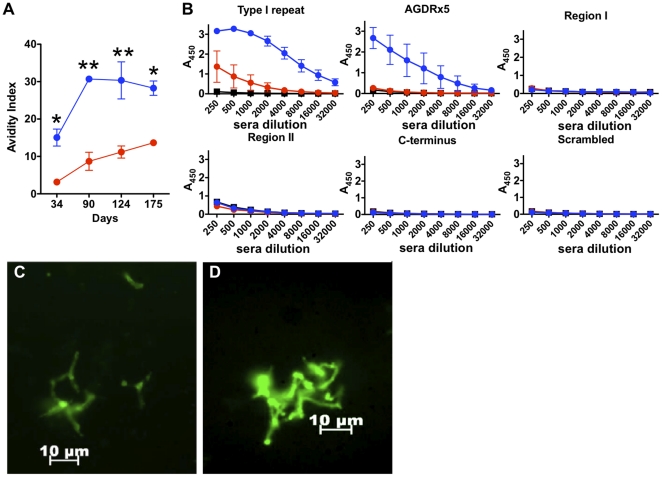
VMP001-NP immunization elicits high avidity antibodies capable of agglutinating live sporozoites. (A) Avidity indices of anti-VMP001 IgG sera obtained from mice immunized as in Fig. 3 with soluble VMP001+MPLA (red circles) or VMP001-NP+MPLA (blue circles) were characterized over 6 months following vaccination. *, *p*<0.01 and **, *p*<0.001, analyzed by two-way ANOVA, followed by a Bonferroni post-test. (B) Anti-VMP001 IgG antibodies elicited with soluble VMP001+MPLA (red circles) or VMP001-NP+MPLA (blue circles) were further examined for their affinities against key fragments of VMP001, including peptides representing the Type I repeat, AGDR motif, Region I, Region II, C-terminus, and scrambled negative peptide control. Sera from non-immunized mice were also included as controls (black squares). (C,D) Sera obtain from mice on day 63 post-immunizations with 2.5 µg VMP001 and 25 µg MPLA in either (C) soluble or (D) VMP001-NP formulations were incubated with live VK210 sporozoites, and immunoflurescence assay was performed to assess recognition of native CSP present on the surface of live sporozoites by anti-VMP001 IgG sera. Mice immunized with VMP001-NP vaccines raised sera that agglutinated live VK210 subtype of *P. vivax*.

Although it is not yet clear which regions of the *P. vivax* CSP confer antibody-mediated protection against sporozoites, several studies thus far have suggested that a humoral response against the Type I repeat is sufficient to induce protection [26], and specifically, the AGDR motif within the VK210 sequence has been identified as the target epitope of a monoclonal antibody that confers protection [27], [28]. We therefore assessed the quality of the antibody responses by measuring the avidity and epitope specificity of sera against the recombinant CSP. The avidity index of anti-VMP001 IgG was determined as the percentage of ELISA antibody titers retained when sera bound to VMP001-coated ELISA plates was exposed to urea, a chaotropic agent that disrupts weakly bound antibodies [22]. VMP001-NP vaccines elicited IgG responses with up to ∼3-fold higher avidity against VMP001 compared to soluble protein vaccination, over the course of 6 months following prime and boost immunizations (*p*<0.001 for day 90 and day 124, **Fig. 6A**).

Serum IgG from mice immunized with VMP001-NP bound the Type I repeat domain with significantly higher titers than soluble VMP001 vaccinations (**Fig. 6B**). In addition, VMP001-NP vaccination elicited antibodies with robust binding to the AGDR motif within the Type I repeat, whereas soluble VMP001 vaccines failed to generate antibodies with the capacity to recognize this protective domain (**Fig. 6B**). Interestingly, all the antibodies generated with these soluble or NP immunizations were directed toward only these two epitopes with minimal recognition of peptides representing N- or C-terminal motifs of the protein. The response to soluble protein vaccines might be altered by higher antigen doses than 1 µg used in this current study, but these results suggest that VMP001-NP+MPLA immunizations generate long-lasting and high-avidity antibody responses with target specificity against sporozoite domains thought to be critical in protective immunity against infection.

### NP immunization raises sera that can recognize and agglutinate live sporozoites

Strong humoral immune responses, characterized by GC formation and durable high-avidity antibody responses indicate that VMP001-NP vaccines are promising candidates for a malaria vaccine. To test whether antibodies generated with lipid-enveloped PLGA vaccines can recognize native CSP antigens, live VK210 *P. vivax* sporozoites were incubated with sera from immunized mice, and IgG bound to the sporozoites was detected by immunofluorescence. Sera from mice immunized with VMP001-NP+MPLA and soluble VMP001+MPLA were both able to bind to the live sporozoites (**Fig. 6C, D**), whereas sera from unimmunized mice failed to bind to the parasites (not shown). Notably, unlike in the group with sera elicited by soluble protein vaccines, live sporozoites incubated with sera from mice immunized with VMP001-NP+MPLA showed extensive agglutination, suggesting that particle-immunized mice elicit antibodies that could potentially render sporozoites inactive and non-infectious [4].

## Discussion

Particulate vaccine carriers can augment immune responses by mimicking structural and compositional aspects of pathogens. Co-delivery of antigens and adjuvant molecules using synthetic particles in the size range of microbes is an efficient and potent way to stimulate CD8^+^ T cell and B cell responses, compared to soluble antigen formulations [17], [29], [30], [31]. We recently showed that nano- or micro-particles prepared with a biodegradable PLGA core and self-assembled surface lipid bilayer promoted strong antibody responses against the model antigen ovalbumin using tiny doses of antigen [19]. Here, we sought to enhance humoral immune responses against a candidate malaria vaccine by delivering the antigen conjugated to the surface membrane of lipid-enveloped PLGA NPs. VMP001-NPs were designed with pathogen-mimicking features, including their virus-like size range (∼300 nm diam.) and membrane composition with phospholipids and immunostimulatory adjuvant, MPLA. To facilitate crosslinking of BCRs and stimulation of B cell responses for strong humoral responses, we conjugated multiple copies of the antigen to each particle membrane, mimicking multivalent display of antigen on viral and bacterial membranes.

Here we have shown that NPs bearing VMP001 and adjuvanted with MPLA elicited significantly higher antibody titers that were long-lasting at lower doses compared to vaccines comprised of soluble protein mixed with MPLA. In addition, compared to soluble formulations, NP vaccines generated a more balanced Th1/Th2 humoral response with significantly enhanced avidity, and required 10-fold less amount of antigen dose to produce similar antibody titers up to 180 days. Notably, compared to vaccination with soluble antigen, NP vaccinations generated antibodies with increased affinities toward the Type-I repeat domain and AGDR motif, which are targets within VMP001 implicated in protective immunity against *Plasmodium* sporozoites [4], [26], suggesting that VMP001-NPs are potently activating B cells with BCR specificity towards VMP001 domains critical for protection against *P. vivax* sporozoites. It is possible that responses to soluble antigen (Th1/Th2 balance, and avidity of humoral responses) could alter as a function of antigen dose, and thus our conclusions are limited by the range of doses explored here. Future experiments will test whether the particle vaccines can elicit and sustain improved humoral immune responses compared to soluble protein vaccines using higher antigen doses and for longer duration than examined in this current study.

The precise mechanism behind the enhanced affinity toward these protective regions by particle vaccine is not known at this point, but we can speculate that multivalent surface-display of VMP001 on the lipid membrane of PLGA NPs may stimulate a more diverse set of naïve B-cells, promoting stimulation of lower avidity cells from the germline antibody repertoire, and enhance affinity maturation during memory B cell development. As a surrogate indicator for protection against malaria sporozoites, we tested the functional efficacy of antibodies using a live sporozoite agglutination assay, and found that the antibodies from mice immunized with VMP001-NPs, compared to soluble protein vaccines, were more efficient at recognition and agglutination of sporozoites, highlighting the potential of NP vaccines to confer protection against malaria infection. Maintenance of high avidity and high titer antibodies against sporozoites may prevent invasion of sporozoites into hepatocytes and initiation of malaria infection, as it has been demonstrated that anti-sporozoite antibodies in dermal tissues can inhibit migration of sporozoites from skin into the systemic circulation via blood vessels [32].

There are two distinct antibody responses after exposure to antigen. Short-lived plasma cells develop in extrafollicular regions in lymph nodes and transiently produce low-affinity antibodies. In contrast, a subset of activated B cells proliferate within follicles to form GCs, where antigen-experienced B cells undergo somatic hypermutation and affinity maturation [33]. These high-affinity B cells then differentiate into long-lived memory B cells or plasma cells, establishing durable memory responses that quickly generate strong humoral immune responses upon re-exposure to antigen [23], [25]. When examined in our model, NP vaccines, but not soluble protein vaccines, induced GC formation even with very low doses of antigen, partially explaining the high-affinity, long-lasting antigen-specific antibody responses elicited with the particle vaccine.

Recent studies have shown that combinations of molecular danger signals can synergize to further boost immune responses when delivered by particulate vaccines [9], [15], [16], [17], [30], [31]. For delivery of multiple immunostimulatory molecules, lipid-“enveloped” PLGA particles allow incorporation of both hydrophilic and hydrophobic molecules in their internal trapped aqueous and polymeric organic phases, respectively. In this current study, an immunostimulatory molecule, MPLA, was added post-synthesis to allow the lipophilic TLR4 agonist to decorate the surfaces of particles [18]. However, lipid “enveloped” PLGA particles offer additional avenues to incorporate adjuvant molecules for their controlled release. Lipophilic adjuvant molecules, such as MPLA, Pam3CysK (TLR-2 agonist), and lipid-conjugated CpG (TLR-9 agonist) recently developed in our laboratory [34], loaded in the organic emulsion phase during synthesis will be partitioned between the lipid layer and polymeric core, allowing their release for an extended period [18]. In addition, hydrophilic adjuvant molecules, such as unmethylated CpG, polyI:C (TLR-3 agonist), and R848 (TLR-7/8 agonist), can also be entrapped in the PLGA core during synthesis [35] or anchored to the membrane via a lipophilic tail [34]. Future studies will be directed to examine the effect of combination of dual or triple adjuvant molecules and their controlled release on the characteristics of humoral immune responses.

In summary, PLGA NPs displaying VMP001 and MPLA on their surfaces potently elicited humoral immune responses against the VMP001 CSP antigen, generating long-lasting antibody responses in mice, characterized by enhanced avidity and capacity to neutralize live sporozoites. NP vaccines thus may confer subunit vaccines with enhanced efficacy against malaria sporozoites.
